# Ebola in West Africa—CDC’s Role in Epidemic Detection, Control, and Prevention

**DOI:** 10.3201/eid2111.150949

**Published:** 2015-11

**Authors:** Thomas R. Frieden, Inger K. Damon

**Affiliations:** Centers for Disease Control and Prevention. Atlanta, Georgia, USA

**Keywords:** Ebola virus, epidemics, West Africa, Centers for Disease Control and Prevention, CDC, viruses, Guinea, Liberia, Sierra Leone

## Abstract

Since Ebola virus disease was identified in West Africa on March 23, 2014, the Centers for Disease Control and Prevention (CDC) has undertaken the most intensive response in the agency’s history; >3,000 staff have been involved, including >1,200 deployed to West Africa for >50,000 person workdays. Efforts have included supporting incident management systems in affected countries; mobilizing partners; and strengthening laboratory, epidemiology, contact investigation, health care infection control, communication, and border screening in West Africa, Nigeria, Mali, Senegal, and the United States. All efforts were undertaken as part of national and global response activities with many partner organizations. CDC was able to support community, national, and international health and public health staff to prevent an even worse event. The Ebola virus disease epidemic highlights the need to strengthen national and international systems to detect, respond to, and prevent the spread of future health threats.

## Abstract 2

Stronger systems are needed for disease surveillance, response, and prevention worldwide.

The unprecedented epidemic of Ebola virus disease (Ebola) in West Africa highlights the need for stronger systems for disease surveillance, response, and prevention worldwide. After a preventable and costly local and global delay, heroic efforts by clinicians and public health personnel and organizations from West Africa and throughout the world broke the cycle of exponential growth of the epidemic and prevented many deaths. As of late 2015, this response, conducted at great expense and personal risk, continues. Here we summarize the experience of the Centers for Disease Control and Prevention (CDC), which complements efforts by the affected countries, the international community, and many partner organizations.

Since Ebola was first reported in West Africa on March 23, 2014, CDC has undertaken the most intensive outbreak response in the agency’s history. As of July 2015, >1,200 CDC employees had deployed to the affected countries for >50,000 person workdays; >3,000 CDC staff, including all 158 Epidemic Intelligence Service Officers, have participated in international or domestic response efforts. For context, over the course of more than a decade, ≈300 CDC staff participated in the smallpox eradication program, one of CDC’s most notable international responses and most intensive technical collaborations with the World Health Organization (WHO) before the current Ebola response (*1*).

CDC had a team of experts on the ground in Guinea within 1 week after the initial case report. When Ebola resurged and spread, CDC activated its Emergency Operations Center (EOC) (*2*) on July 9, 2014. Since then, CDC has coordinated >1,400 deployments to Guinea, Liberia, and Sierra Leone and sent staff to help Nigeria (*3*), Senegal (*4*), and Mali (*5*) prevent the spread of Ebola. CDC staff also have undertaken development of new diagnostic tests (*6*) and research to evaluate therapeutic drugs (*7*) and vaccine efficacy (*8*,*9*). As of mid-2015, >500 CDC staff continued working throughout the 3 most heavily affected nations (Guinea, Sierra Leone, and Liberia), the West Africa region, and the United States.

At the peak of the epidemic in fall 2014, widespread transmission of Ebola virus was occurring in the capitals of Liberia and Sierra Leone; health care systems had become largely nonfunctional; Ebola cases or clusters occurred in other countries of Africa; and there was a real possibility that Ebola could spread widely and become endemic in some of the poorest and sickest countries of the world. As of late2015, although the region is not Ebola-free, enormous progress has been made. There is a risk for resurgence and cross-border spread, and because the status of Ebola virus reservoirs is not confirmed and the possibility of sexual transmission from survivors persists, the potential exists for periodic outbreaks.

## CDC Response in Heavily Affected Countries

### Incident Management

One challenge in responding to complex outbreaks is coordination among partners. CDC’s priority in West Africa during summer 2014 was to augment the efficiency of response activities through incident management systems run by national leaders and supported by an EOC reporting to the president of each affected country. These systems were developed in collaboration with WHO and served as the focal point for international assistance. CDC also helped countries establish subnational EOCs in areas with Ebola virus transmission in Liberia and Guinea; the United Kingdom similarly played a key role in Sierra Leone. When resources had to be mobilized rapidly, the CDC Foundation, a not-for-profit philanthropic entity authorized by the US Congress in 1992 to help CDC improve its response capacity (*10*,*11*), supported staffing, logistics, data management, informatics, and operations of EOCs.

### Epidemiology and Surveillance

Working with governments, nongovernmental organizations, and WHO, CDC epidemiologists assisted national- and district-level staff in each country in identifying cases and contacts and trained in-country staff to perform these essential public health activities. Because clinical, public health, laboratory, and data systems were overwhelmed (*12*), CDC staff assisted with data entry and management, including geographic information systems to track and evaluate disease trends.

### Contact Tracing

After the cycle of exponential epidemic growth was broken and personnel could refocus on contact identification, CDC strengthened work with national counterparts and WHO to help improve the quality of contact identification and follow-up, including isolation of symptomatic contacts for clinical assessment and laboratory testing. These activities were vital to reduce Ebola transmission. WHO has played a critical role in improving contact tracing and contact management, particularly in Guinea (*13*).

### Laboratory Testing

Global collaboration with laboratories from a European Union consortium made real-time quantitative reverse transcription PCR available in the heavily affected West Africa countries for patients and decedents suspected of having Ebola. CDC experts helped coordinate the laboratory section of the incident management system, supported laboratories in Liberia with the US Department of Defense (DoD) and National Institutes of Health, and operated a field laboratory in Bo, Sierra Leone, that processed >2,000 samples during a 3-week period at the height of the epidemic (*14*); by mid-2015, that laboratory had processed >20,000 samples.

### Rapid Isolation and Treatment of Ebola Patients

**Figure 1:**
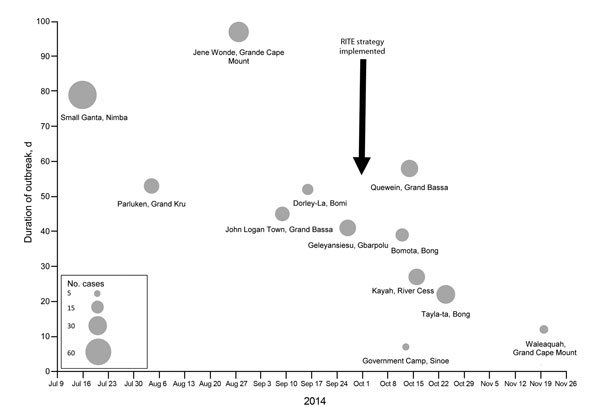
Decreased size and duration of outbreaks in remote areas before and after implementation of the Rapid Isolation and Treatment of Ebola (RITE) strategy, Liberia, 2014. Size of circle is proportional to number of cases in cluster.

Rapid isolation and treatment of Ebola patients is a key strategy to stop Ebola outbreaks. Each country had limited capacity to isolate and treat patients, and strategies to do so effectively and safely evolved over time. In collaboration with the US Agency for International Development’s Office of Foreign Disaster Assistance (USAID/OFDA), WHO, DoD, and multiple other partners, CDC provided technical support and training to establish Ebola treatment units (ETUs) and community care centers. Beginning in early October 2014, CDC designed and helped implement a strategy of rapid isolation and treatment of Ebola (RITE) in Liberia. This strategy controlled outbreaks faster and supported the care of patients in remote areas, cutting the time to control outbreaks in half (Figure 1) and doubling survival rates (*15*).

### Infection Control

In the 3 heavily affected countries, CDC and its partners trained >25,000 health care workers in infection control, including use of personal protective equipment (PPE) (*16*). A 3-day hands-on training course designed by CDC with Médecins Sans Frontières trained >600 US health care providers on Ebola clinical care and infection control before their deployment to West Africa (*17*).

### Health Promotion and Communications

In addition to the efforts of partner organizations, CDC field teams included emergency risk communication specialists to generate and disseminate accurate information, address rumors, decrease stigma, reduce unsafe burial practices, and respond to community needs. CDC staff in Liberia and Sierra Leone identified and promulgated burial practices that met community needs for culturally acceptable mourning, thus reducing resistance to safe burials (*18*,*19*). In all countries, community engagement and effective communication were key strategies for successful outbreak control.

### Technical Guidance

CDC has issued >200 scientific documents, including >100 technical guidance documents, covering many aspects of the response. CDC staff also worked closely with UNICEF and other partners to develop guidance in related areas, such as safe reopening of schools (*20*).

### Mobilization of Partners

During summer 2014, CDC recognized that despite Médecins Sans Frontières’ massive response; CDC’s own response; and responses of affected countries, WHO, and international partners, the epidemic was spiraling out of control. CDC then advocated to increase involvement by the US government and the global community.

DoD, along with USAID/OFDA’s Disaster Assistance Response Team (DART), has been a key partner in this scale-up. Initially focused on researching treatments and vaccines and providing laboratory diagnostics, in September 2014, DoD took the lead on constructing, supplying, and maintaining a field hospital to treat health care workers with Ebola in Liberia. DoD also deployed 3,000 military personnel for logistics and coordination, provision of medical personnel to train health care workers, establishment of additional treatment centers in Liberia, and operation of 3 mobile medical laboratories (*21*). The DART provided coordination to rapidly engage partners providing services and supporting response efforts; CDC staff served as the technical lead for health, public health, and medical issues within the DART.

### Epidemic Modeling

A CDC model that projected the possible trajectory of the epidemic if the trend of rapid transmission through August 2014 continued unabated was key to increasing the speed and scale of the US and global response (*22*). The worst-case scenarios of the model made clear the need for urgent action and helped stimulate a massive global response.

Analysis from the model provided 4 key findings. First, cases were increasing exponentially, and the response needed was massive and urgent. CDC helped facilitate assistance, including from the African Union, which mobilized nearly 1,000 staff, including doctors, nurses, epidemiologists, and health educators (*23*).

**Figure 2:**
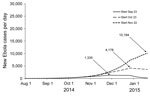
Estimated impact of delaying intervention on daily number of Ebola virus disease cases, Liberia, 2014–2015. The intervention modeled is as follows: starting on September 23, 2014 (day 181 in model), and for the next 30 days, the percentage of all patients in Ebola treatment units increased from 10% to 13%. This percentage was again increased on October 23, 2014 (day 211 in model) to 25%, on November 22, 2014 (day 241 in model) to 40%, and finally on December 22, 2014 (day 271 in model) to 70%. Day 1 in model is March 3, 2014. The impact of a delay of starting the increase in interventions was then estimated by twice repeating the above scenario but setting the start day on either October 23, 2014, or November 22, 2014. When the intervention is started on November 22, 2014, the peak is not reached by January 20, 2015, which is the last date included in the model. Graph based on Figure 10 in Meltzer et al. (*22*).

Second, the model predicted a severe penalty for delay; case numbers at the peak roughly tripled for every month of delayed scale-up (Figure 2). Thus, interventions (isolation, treatment, and safe burials) had to be rapid, with action and progress measured in hours and days rather than in weeks and months. In each country, CDC encouraged national leaders, incident managers, health workers, the media, and communities to take action, immediately because even a rapid international response would not be fast enough.

Third, the model identified a tipping point at which the epidemic would plateau and decline if enough (i.e., >70%) Ebola patients were isolated effectively and decedents buried safely. This finding led to establishment of community isolation facilities (*24*) and to contracting by USAID/OFDA for burial teams that worked to technical specifications established by CDC, first in Liberia and later in Sierra Leone (*25*). In Liberia, experienced CDC public health specialists conducted detailed planning exercises with community, political, medical, and public health leaders in each county to identify where sick persons could be isolated until ETUs were constructed and how contacts could be monitored and cared for if they became ill.

**Figure 3:**
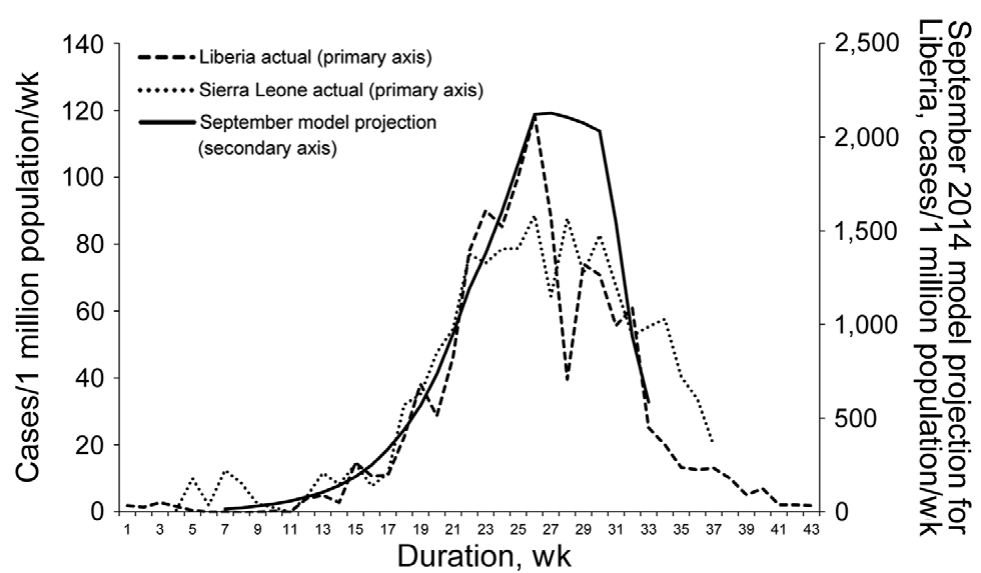
Comparison of estimated weekly Ebola virus disease case rate for Liberia with intervention with actual weekly case rates for Liberia and Sierra Leone. The September 2014 modeled projection curve was based on Figures 9 and 10 in Meltzer et al. (*22*), by using model predictions calculated assuming that interventions started on September 24, 2014. Liberia, week 1 begins May 4, 2014; Sierra Leone, week 1 begins May 25, 2014. The model projected the incidence that would occur if the proportion of Ebola patients who were hospitalized was 25% at week 22, increased to 40% at week 26, and increased again to 70% at week 30, while the proportion in effective home isolation remained constant at 10%. The similarity in the increase and decrease in the actual epidemic curves in both Sierra Leone and Liberia closely match the model after taking into account differences in start dates and population sizes between the 2 countries, implying that the proportion of cases effectively isolated in both countries followed a similar time course as the model.

**Figure 4:**
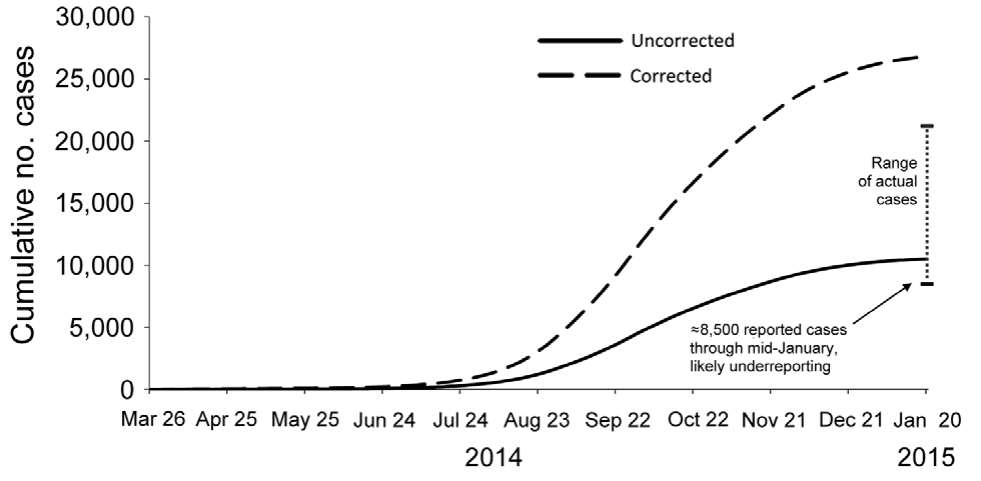
Comparison of the estimated impact of interventions on number of Ebola cases with actual cases reported, Liberia, 2014–2015. The September 2014 modeled projection curve was based on Figure 3 in Meltzer et al. (*22*) by using model predictions calculated assuming that interventions started on September 24, 2014. The corrected curve of projected cases is adjusted for potential underreporting by multiplying reported cases by a factor of 2.5. Actual reported cases are from World Health Organization situation report for January 21, 2015 (*26*).

Fourth, the model predicted that when the tipping point was reached, transmission would decline rapidly. This prediction was shown to be accurate in the following months in Liberia and Sierra Leone (Figure 3). For Liberia, the model’s prediction that if urgent action were taken, there would be 10,000–27,000 cumulative cases by January 21, 2015, closely matched the 8,500–24,000 cases that occurred (Figure 4). The predictions also closely matched the actual case trajectory after effective intervention.

### Border Health Security

CDC worked with ministries of health and airport authorities in all 3 heavily affected countries, as well as in other affected countries, to establish screening of travelers leaving the country by air to prevent sick or exposed persons from boarding planes. By mid-2015, >200,000 travelers leaving Guinea, Liberia, and Sierra Leone had been screened. In addition to reducing the likelihood of additional spread of Ebola to other countries, this screening, along with CDC’s work with airlines to address air transport industry and flight crew concerns, helped enable humanitarian and public health organizations to sustain travel to affected areas by regular commercial airline flights. CDC staff also provided technical assistance on measures to reduce risk for spread through maritime ports and across land borders.

### Innovation

CDC laboratory scientists implemented high-throughput laboratory capacity by using robotics and collaborated with private industry to promote development of lateral-flow assays to detect Ebola in point-of-care settings within 30 minutes after a finger stick or oral swab (*6*). In addition to supporting the National Institutes of Health randomized controlled trials of Ebola treatment (*27*) and vaccines (*28*), CDC staff worked with Sierra Leone authorities to implement a parallel Sierra Leone Trial to Introduce a Vaccine against Ebola (STRIVE), an adaptive, phased-introduction trial of a vaccine candidate among health workers in that country (*8*,*9*).

## Support to Other At-Risk Countries

In Nigeria, a cluster of Ebola cases in July 2014 resulted from a traveler from Liberia. CDC deployed disease control experts to Lagos, the country’s most populous city, within 72 hours and, in the first week after disease confirmation, supplemented response efforts with 13 Field Epidemiology Training Program (FETP) trainees, graduates, and trainers who had experience in epidemiology and infection control. In the 2 weeks that followed, CDC sent additional agency staff and helped mobilize 40 CDC-trained physicians from Nigeria’s FETP. With the Nigerian government and partners, CDC facilitated creation of an effective incident management system, using leadership and staff from the Nigerian Polio Eradication Program and support from the Bill and Melinda Gates Foundation. This incident management system oversaw training of 2,300 health care staff, creation of an ETU in 14 days, and identification of >800 contacts; conducted 19,000 home visits of these contacts to monitor symptoms and temperatures; and screened >150,000 persons at airports. Although 19 secondary cases of Ebola occurred in 3 generations of spread in 2 cities, this rapid action controlled transmission, and Nigeria has been Ebola-free since this incident (*3*).

CDC staff provided similar assistance in Mali after a child arriving from Guinea died of Ebola and again after a cluster of cases occurred from a person from the Mali–Guinea border who had previously undiagnosed Ebola (*29*), and in Senegal after an incident of disease importation (*4*). CDC also collaborated with WHO to increase preparedness in at-risk countries by helping establish EOCs, surveillance for hemorrhagic fever and clusters of deaths, training in contact tracing, laboratory specimen transport and testing, isolation capacity for patients suspected of having Ebola, health communication messages, and border health security.

## Ebola in the United States

Before diagnosis of the first case of Ebola imported to the United States, CDC alerted US health care providers to consider Ebola if compatible signs and symptoms manifested within 21 days after a traveler arrived from an affected country (*30*). CDC also issued infection control guidance for hospitals (*31*); strengthened laboratory networks and existing surveillance systems; and disseminated recommendations for travelers on the CDC website, through social media channels, and at US international airports.

The first case of Ebola diagnosed in the United States, imported by a traveler from Liberia, revealed gaps in hospital preparedness and response capabilities (*32*). Ebola was not considered in the patient’s initial presentation, despite fever and travel history to Liberia. CDC provided assistance to the state and local health departments and to nearby hospitals. Two nurses caring for the patient were infected, most likely as the result of underprepared processes, lack of training, and suboptimal use of PPE during the first few days of the patient’s second hospitalization, before his Ebola diagnosis. CDC subsequently strengthened recommendations for infection control, particularly training, supervision, and specifications of PPE. The second nurse who became ill was allowed to travel by air despite exposure that CDC should have categorized as high-risk to prevent the nurse from flying (*33*). In turn, this measure would have reduced the number of travelers whose health was monitored and the work of public health personnel monitoring contacts.

Recognizing a need for enhanced preparedness and training, CDC staff then visited 81 facilities in 21 states and Washington, DC, helping 55 of these facilities qualify as Ebola Treatment Centers for patients with suspected or confirmed Ebola. CDC also has qualified 56 state, county, and local public health laboratories to perform real-time quantitative reverse transcription PCR for Ebola with a Food and Drug Administration–approved DoD assay developed by the US Army Medical Research Institute of Infectious Diseases (*34*).

CDC established Ebola Response Teams composed of CDC experts in infection control, clinical care, contact tracing, communications, environmental waste management, and other areas to support state and local health departments and to deploy to any hospital in the United States that has a patient under investigation for Ebola (*35*). CDC staff arrived at New York City’s Bellevue Hospital before Ebola was confirmed in the patient treated there.

To strengthen protection throughout the United States and to preclude restrictions on travel that could have undermined the response in West Africa and led to surreptitious travel from the region, CDC, together with the US Customs and Border Protection and state and local public health departments, developed a postarrival monitoring program to educate and follow >20,000 travelers arriving in the United States from Guinea, Liberia, and Sierra Leone since October 2014 (*36*). Travelers are met at the airport and provided with Check and Report Ebola (CARE) kits that include health education materials, a thermometer, and ways to connect with their state or local health department, including a prepaid cell phone. Through mid-May 2015, >1,200 travelers were referred to CDC for additional screening because of illness or, more commonly, to assess possible exposures; 28 persons were referred for medical evaluation. Ebola was not diagnosed in any of these persons (*37*).

Nearly 500 persons considered to be at “some or high risk” received direct active monitoring that included daily direct observation of symptoms and temperature monitoring by health workers. More than 20,000 travelers classified as “low but not zero risk” received active monitoring, in which they monitored their own temperature and any symptoms and reported daily to the state or local health department until 21 days after their departure from an Ebola-affected country (an effort that has involved >400,000 cumulative contacts with arriving travelers). Health departments facilitated safe transport to a hospital ready to assess travelers for Ebola if the person developed fever or other symptoms of concern.

Before initiation of the active monitoring program, 1 case of Ebola was detected by self-monitoring; rapid detection and isolation prevented further disease transmission. Every jurisdiction now monitors travelers arriving from the highly affected countries and reports to CDC.

## Lessons

The Ebola epidemic in West Africa is unprecedented in size and geographic distribution; it spread in many areas unfamiliar with the disease, including the first large urban outbreaks of Ebola. If the response in West Africa and global assistance had been implemented earlier, faster, and more effectively, far fewer cases and deaths and much less social and economic disruption would have occurred. The epidemic has shown that critical improvements are needed in 2 main areas. First, the ability of every country to quickly identify and respond to a health threat needs to be enhanced. Second, the ability of the global community to rapidly respond to needs in a country overwhelmed by an epidemic must be improved.

For months, the Ebola epidemic spread faster than the international community, including CDC, responded. Critical barriers in the affected countries include limited electronic connectivity (*38*); insufficient numbers of trained staff; inability to surge rapidly enough to provide needed case detection, education, contact tracing, and isolation services; and poorly functioning national health and public health systems with staff who often were unpaid, untrained, and poorly supervised. Surveillance and data management systems were overwhelmed; solutions are needed to manage, track, and support large outbreaks and public health interventions.

Stronger national and international systems for disease detection and control are needed. Paradoxically, the world is better prepared to find and stop emerging health threats than at any time in history, yet also is at greater risk for rapid spread of infectious diseases, which occur more frequently because of encroachment into forest areas, spread of antimicrobial-resistant organisms, and increasing ease of creation of dangerous pathogens, in the context of an increasingly mobile, interconnected, and urban world. The global community must use these lessons to improve response systems for large-scale emergencies, following the principles of the International Health Regulations, while using core staff and facilities on a daily basis to respond to ongoing health problems.

If the 3 highly affected countries had had effective surveillance and containment systems in place before 2014, the outbreaks might have been detected and stopped promptly (*39*). There was an unrecognized need for more effective control in urban areas with mobile populations. The use of the incident command system in this complex scenario was critical for organizing focused efforts to stop chains of transmission at the community level and within the health care system. Trust and coordination had to be established with more diverse communities, many of which were in postconflict environments, than in past outbreaks. In all 3 countries, emergency risk communication was a dynamic process, changing as the outbreak evolved, to promote understanding of nuanced messages of risk. Community engagement and understanding of each local community’s beliefs and traditional practices was critical to success of the overall response and particularly important to ensure rapid isolation of infected patients, complete elicitation and monitoring of contacts, and safe burials.

In Uganda, where CDC and others have invested in public health for years, cases of Ebola and Marburg virus disease are now diagnosed promptly, infection control and contact tracing quickly implemented, and outbreaks either stopped rapidly or prevented altogether (*40*). Similarly, leveraging infrastructure and assets developed through the polio eradication efforts in Nigeria enabled an effective rapid response and demonstrated the value of investing in core public health capacities and training epidemiologists through the country’s FETP program, which is needed in countries around the world. In contrast, before the outbreak, CDC had limited activities and no offices in any of the 3 heavily affected countries. The Global Health Security Agenda, supported by the United States in partnership with other nations and international organizations, seeks to rapidly improve the capacity of countries throughout the world to find, stop, and, wherever possible, prevent the spread of health threats (*41*,*42*).

Sustainable response capacity of international entities also needs to be improved. There were initial delays in effective response by WHO country offices and initial resistance of these offices and the African Region of WHO to involve CDC and other organizations (*43*). WHO has since mounted an effective response supporting the core public health interventions to stop spread of Ebola and is working to become more effective. The Global Outbreak Alert and Response Network is designed to provide a global response (*44*) but needed more staff with a wider range of skills to be deployed rapidly and for longer periods of time. Organizations that participated in the response needed a broad range of skills, including expertise not only in laboratory and epidemiologic functions but also clinical care, logistics, health communications, information technology, data management, and anthropology, as well as fluency in English, French, and local languages and substantial knowledge of the cultural, social, and religious sensitivities that need to be addressed to engage communities and stop the spread of disease. CDC, along with communities, health care workers, and leaders in the affected nations and the international community, will continue to respond until the Ebola epidemic ends and is committed to strengthening national capacities in West Africa and elsewhere to prevent similar epidemics in the future.
